# TRPV2 modulates mechanically Induced ATP Release from Human bronchial epithelial cells

**DOI:** 10.1186/s12931-024-02807-0

**Published:** 2024-04-27

**Authors:** Orla M. Dunne, S. Lorraine Martin, Gerard P. Sergeant, Daniel F. McAuley, Cecilia M. O’Kane, Brian Button, Lorcan P. McGarvey, Fionnuala T. Lundy

**Affiliations:** 1 Wellcome-Wolfson Institute for Experimental Medicine, School of Medicine, Dentistry and Biomedical Sciences, Queen’s University Belfast, 97 Lisburn Road, Belfast, BT9 7BL UK; 2 School of Pharmacy, Queen’s University Belfast, Belfast, UK; 3 Smooth Muscle Research Centre, Dundalk Institute of Technology, Co. Louth, Dundalk, Ireland; 4 Department of Biochemistry and Biophysics, University of North Carolina at Chapel Hill, Chapel Hill, NC USA

**Keywords:** Transient receptor potential channels, Mechanotransduction, Adenosine triphosphate, Purinergic P2 × 3

## Abstract

Repetitive bouts of coughing expose the large airways to significant cycles of shear stress. This leads to the release of alarmins and the tussive agent adenosine triphosphate (ATP) which may be modulated by the activity of ion channels present in the human airway. This study aimed to investigate the role of the transient receptor potential subfamily vanilloid member 2 (TRPV2) channel in mechanically induced ATP release from primary bronchial epithelial cells (PBECs).

PBECs were obtained from individuals undergoing bronchoscopy. They were cultured in vitro and exposed to mechanical stress in the form of compressive and fluid shear stress (CFSS) or fluid shear stress (FSS) alone at various intensities. ATP release was measured using a luciferin–luciferase assay. Functional TRPV2 protein expression in human PBECs was investigated by confocal calcium imaging. The role of TRPV2 inhibition on FSS-induced ATP release was investigated using the TRPV2 inhibitor tranilast or siRNA knockdown of TRPV2. TRPV2 protein expression in human lung tissue was also determined by immunohistochemistry.

ATP release was significantly increased in PBECs subjected to CFSS compared with control (unstimulated) PBECs (*N* = 3, ***P < 0.001). PBECs expressed functional TRPV2 channels. TRPV2 protein was also detected in fixed human lung tissue. ATP release from FFS stimulated PBECs was decreased by the TRPV2 inhibitor tranilast (*N* = 3, **P < 0.01) (vehicle: 159 ± 17.49 nM, tranilast: 25.08 ± 5.1 nM) or by TRPV2 siRNA knockdown (*N* = 3, *P < 0.05) (vehicle: 197 ± 24.52 nM, siRNA: 119 ± 26.85 nM).

In conclusion, TRPV2 is expressed in the human airway and modulates ATP release from mechanically stimulated PBECs.

**Supplementary Information:**

The online version contains supplementary material available at 10.1186/s12931-024-02807-0.

## Introduction

During bouts of cough, the airway lumen is narrowed to approximately one-fifth of its original diameter [1] and together with the shear stress created by high expiratory flow rates, the mechanical forces exerted on the airway wall are substantial [2]. In COPD and chronic cough, where patients report frequent bouts of coughing, the inflammatory consequences of repeated mechanical stimulation of the bronchial epithelium may be associated with airway remodelling and structural change [3] representing a distinct contribution to the pathophysiology of these conditions.

In response to mechanical stimulation, bronchial epithelial cells release a range of endogenous inflammatory molecules in the form of small molecules, including extracellular adenosine triphosphate (ATP), inflammatory cytokines and secrete enzymes such as matrix-metalloproteinases [4–8]. Increased ATP release has been recorded following fluid shear stress (FSS) treatment of bronchial epithelial, alveolar and adenocarcinoma cell lines [4–6], . The upregulation in production of the inflammatory cytokine interleukin-8 (IL-8) from the BEAS-2B bronchial epithelial cell line has been reported with stretching mechanical forces [7]. Likewise, increased activation of matrix metalloproteinase family members was reported in bronchial epithelial cells with compressive stress [8]. Recent interest has focused on the mechanisms responsible for ATP release, particularly during bouts of coughing. Increased ATP levels have been reported in airway lavage samples from patients with COPD which correlate with disease severity [9]. There is also evidence that ATP promotes the migration of immune cells to inflammatory sites and the release of chemokines and growth factors [10]. Furthermore, ATP may itself exert tussive effects, with evidence suggesting that inhaled ATP evokes more cough in individuals with COPD compared to healthy individuals [11]. In refractory and unexplained chronic cough, results from recent phase 3 clinical trials of Gefapixant (which blocks the ATP-gated purinergic receptor, P2 × 3) support a role for ATP as an intrinsic component in cough hypersensitivity [12].

ATP release from cells occurs through five primary routes including vesicular-mediated release, pannexin channels, connexin hemichannels, volume-regulated anion channels and maxi-anion channels [13]. In addition, auxiliary ATP release channels include members of the transient receptor potential (TRP) superfamily of ion channels [13–17]. Certain members of the TRP superfamily act as mechanosensors; including TRP subfamily vanilloid member 2 (TRPV2) which has a role in mechanotransduction of stretch in cardiac muscle and in retinal arterioles [18, 19]. However, the role of TRPV2 in mechanosensation of the human airway has not previously been studied, and the potential for TRPV2 to function as an auxiliary ATP release channel is unknown.

In this study, we investigated the hypothesis that TRPV2 has a modulatory role in ATP release from mechanically stimulated human bronchial epithelial cells.

## Materials and methods

### PBEC culture

PBECs were established from bronchial brush samples obtained from individuals undergoing routine bronchoscopy with ethical approval from the NHS Health Research Authority, East of England - Cambridge East Research Ethics Committee (REF 18/EE/0048) or from ex-vivo human lung tissue with ethical approval by UK Research Ethics Committee (REF 14/LO/0250). PBECs were established in Pneumacult^TM^-ex plus medium (StemCell Technologies, Vancouver, Canada) and on reaching 50 – 60% confluence, sub-cultured using animal component-free cell dissociation kit (StemCell Technologies). A total of 5 PBEC samples were used throughout this study (PBEC_1 to PBEC_5), with donor gender, obstructive phenotype, smoking history FEV_1_ (% predicted) and FEV_1_ available in Table E1.

### Mechanical stress treatment of PBECs

**Fig. 1:**
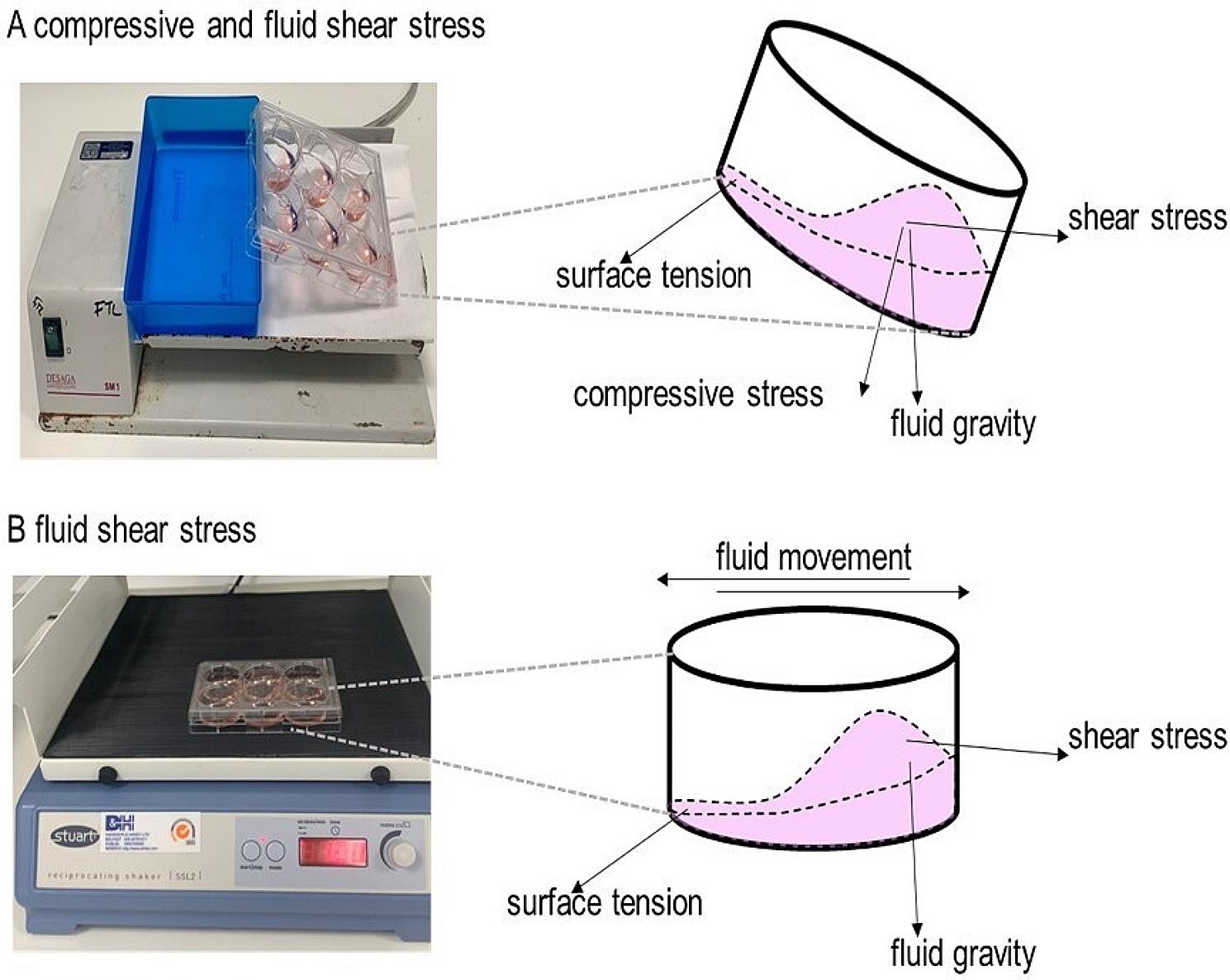
Mechanical Stimulation of PBECs Implemented Using a Seesaw Rocking Shaker or a Reciprocating Shaker. To evoke compressive and fluid shear stress (CFSS) a 6-well culture plate was placed on the platform of a seesaw rocking shaker (Sarstedt, Nümbrecht, Germany). Briefly, 6-well plates were placed on the seesaw rocking shaker and titled at a 30° angle horizontal to the ground. The plate was rotated from left to right every 15 s for 15 min, after which point the direction of the 30° angle of the 6-well plate was alternated and the rotation of the plate from left to right every 15 s recommenced for a further 15 min. Movement of the platform induces a wave of culture medium that settles at the bottom of the well for 15 s before a wave in the other direction occurs. This form of stimulation results in fluid shear stress, compressive stress and liquid surface tension from the thin layer of cell culture medium at the upper side of the well and fluid gravity being applied to the cells at the base of the well (**A**). To evoke fluid shear stress (FSS) 6-well culture plate was placed on the platform of a reciprocating shaker (Avantor, Pennsylvania, USA). The 6-well plate was stimulated with 0 (control), 25, 50 or 100 cycles per minute (CPM). Movement of the platform induced a wave of culture medium, stimulating cells with fluid shear stress stress and liquid surface tension from the thin layer of cell culture medium at one side of the well and fluid gravity being applied to the cells at the other side of the well (**B**)

In the absence of a consensus on a gold standard method to replicate airway wall shear stress in vitro we utilised two methods of mechanical stress to stimulate PBECs prior to measuring ATP release. The oscillatory compressive and fluid shear stress (CFSS) method of mechanical stimulation was achieved by using a seesaw rocking shaker [4] that incorporated compressive stress from the application of culture medium fluid gravity to PBECs at the base of the well in addition to fluid shear stress from the culture medium movement above PBECs (Fig. 1a). However, this combined model did not allow flexibility to adjust the mechanical stimulus intensity to evaluate a dose response for mechanical stress intensity and ATP release. To overcome this, we developed an in-house method of mechanical stimulation allowing oscillatory fluid shear stress (FSS) exposure at various intensities using a reciprocating shaker to achieve FSS from the movement of the layer of culture medium above PBECs with forces of 0 cycles per minute (CPM), 25 CPM, 50 CPM or 100 CPM (Fig. 1b) inducing approximate forces of 0.09, 0.18 and 0.41 dynes/cm^2^ respectively on the cells (calculations provided in online supplement). Cultured PBECs were subjected to mechanical stimulation for 30 min, and ATP in conditioned medium was measured immediately by ATPlite luciferin-luciferase assay (Perkin Elmer, Llantrisant, UK). To assess if mechanical stress induces the increased release or activity of other inflammatory mediators in addition to ATP, IL-8 release and MMP-13 activity was measured in conditioned medium or cell lysate respectively 24 h after PBEC mechanical stimulation by ELISA (R&D systems, Abingdon, UK) or fluorescence resonance energy transfer assay (SensoLyte ® Plus 520; AnnaSpec, Fremont, USA) respectively.

### Assessing the expression and role of TRPV2 in ATP release from PBECs

The expression of TRPV2 in cultured PBECs and human lung tissue was carried out using immunocytochemistry and immunohistochemistry respectively (detailed protocols are provided in the online supplement). To assess functional TRPV2 responses, intracellular calcium measurements in PBECs were obtained by confocal calcium imaging with the TRPV2 agonist cannabidiol (CBD) [1 µM] and the TRPV2 inhibitor tranilast [1 µM]. Further details are provided in the online supplement. For experiments investigating the role of TRPV2 in ATP release, PBECs were treated with tranilast [1 µM] or vehicle control (DMSO) 30 min prior to mechanical stimulation and ATP was measured immediately by ATPlite luciferin-luciferase assay. For additional experiments investigating the role of TRPV2 in ATP release, PBECs were transfected with 0 or 5 nM TRPV2 siRNA (Qiagen predesigned NM_016113, Venlo, The Netherlands) or scrambled control siRNA (Qiagen, 1,027,281) using HiPerfect transfection reagent (Qiagen) 24 h after seeding onto 6-well plates. PBECs were treated with FSS mechanical stimulation 48 h after transfection and ATP was measured immediately by ATPlite luciferin-luciferase assay.

### Statistical analysis

Summary data are presented as mean ± standard error of the mean (SEM). Statistical comparisons were performed using Prism V9.3.1 (GraphPad Software, San Diego, USA). Data were analysed using statistical tests outlined in the relative figure legends with *P* < 0.05 considered statistically significant.

Materials and methods are described in detail in the online supplement.

## Results

### ATP release from mechanically stimulated PBECs

**Fig. 2:**
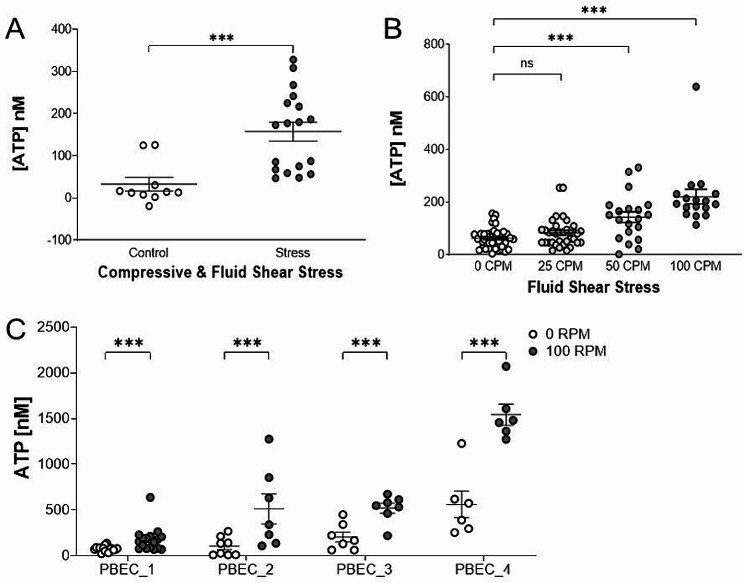
Measurement of ATP in Mechanically Stimulated Primary Bronchial Epithelial Cells (PBECs). ATP release from control and mechanically stressed PBECs using compressive and fluid shear stress treatment (CFSS) was measured with the ATPlite luciferin-luciferase assay. Mean with SEM, *N* = 3 independent experiments. Mann Whitney test, ***P < 0.001 (**A**). ATP release from control (0 CPM) and mechanically stressed PBECs using fluid shear stress (FSS) at 25, 50 and 100 CPM was measured with an ATPlite luciferin-luciferase assay. Mean with SEM, *N* = 3 independent experiments. Kruskal-Wallis test with Dunn’s multiple comparisons test, ***P < 0.001 (**B**). PBECs originated from a single donor (PBEC_1) (**A** and **B**). ATP release from control (0 CPM) and FSS stimulated PBECs (100 CPM) were measured with an ATPlite luciferin-luciferase assay. Mean with SEM, *N* = 3 independent experiments. Two-way ANOVA with Tukey’s multiple comparisons post-test, ***P < 0.001. PBECs originated from 4 individual donors (PBEC_1, PBEC_2, PBEC_3 and PBEC_4) with various phenotypes and smoking histories (**C**)

ATP release from PBECs treated with CFSS was significantly increased (*P* < 0.001) compared to non-stressed (control) PBECs (stress: 157 ± 22.17 nM, control: 32.89 ± 15.9 nM) (Fig. 2a). ATP release from FSS-stimulated PBECs was significantly increased compared to control PBECs (0 CPM vs. 50 CPM and 100 CPM: *P* < 0.001) dependent on the intensity of the mechanical stimulus applied to the cells (0 CPM: 62.41 ± 5.35 nM, 25 CPM: 83.25 ± 9.1 nM, 50 CPM: 143 ± 19.86 nM, 100 CPM: 221 ± 27.95 nM) (Fig. 2b). Using the FSS method of mechanical stimulation, we observed a comparable concentration of ATP released at the submaximal intensity (50 CPM) to the concentration of ATP released from the CFSS method. In contrast, we saw no increase in IL-8 or MMP-13 levels following FSS stimulation (supplementary Fig. 1a and 1b).

Results showing significantly increased (*P* < 0.001) ATP release from FSS stimulated PBEC_1 at 100 CPM compared to control PBECs were confirmed in PBECs from three additional donors with various obstructive phenotypes and smoking histories (PBEC_2 (100 CPM: 512 ± 164 nM, 0 CPM: 102 ± 36.04 nM), PBEC_3 (100 CPM: 519 ± 55.8 nM, 0 C: 205 ± 54.98 nM) and PBEC_4 (100 CPM: 1543 ± 116 nM, 0 CPM: 560 ± 146 nM)) (Fig. 2c). Mechanical stimulation of PBECs stimulated with either CFSS or FSS did not result in a decrease of cell viability as measured by 3-(4,5-dimethylthiazol-2-yl)-2,5-diphenyltetrazolium bromide (MTT) assay (supplementary Fig. 2a and 2b).

### PBECs Express Functional TRPV2

**Fig. 3:**
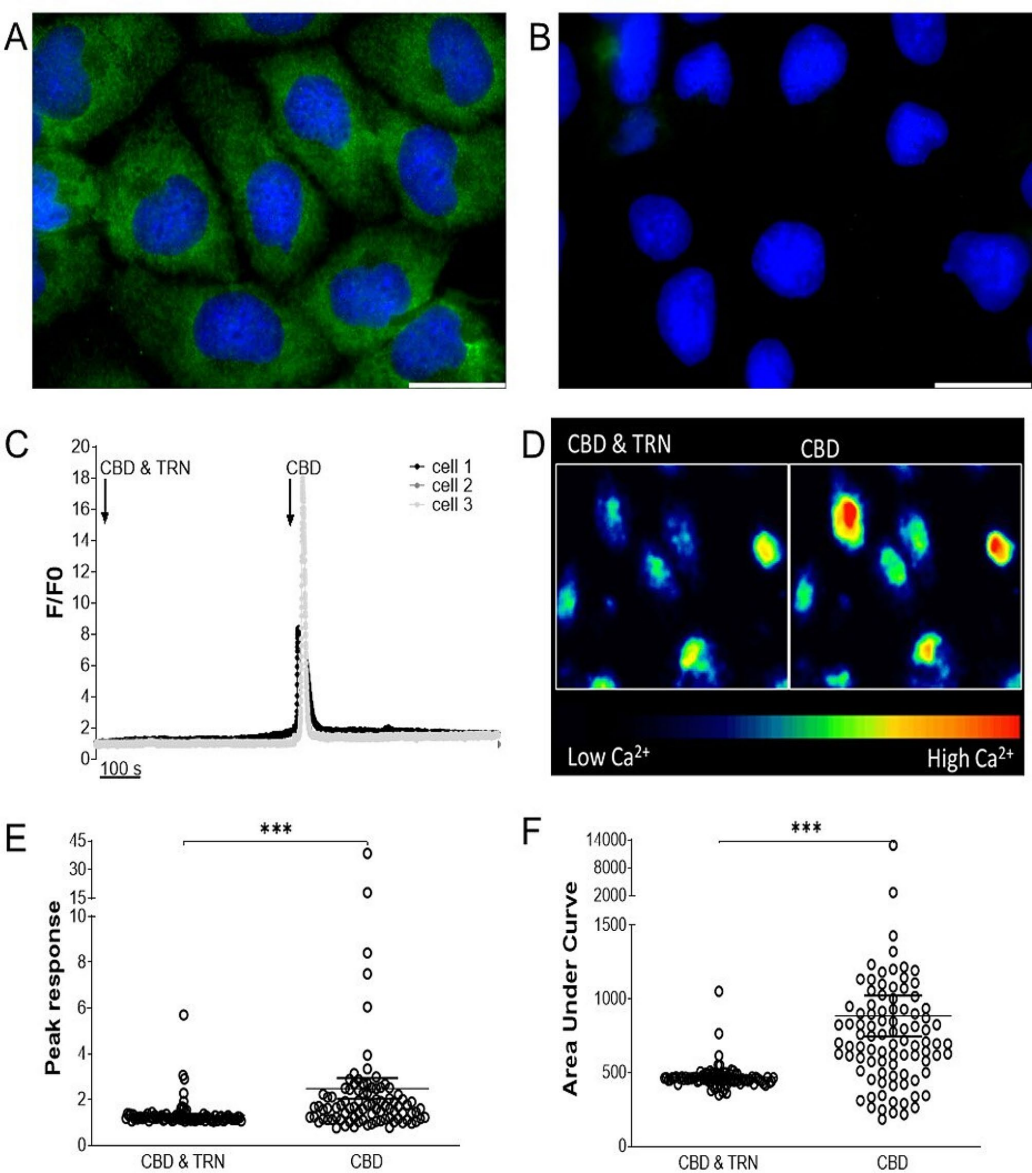
TRPV2 Functional Expression in Primary Bronchial Epithelial Cells (PBECs). PBEC_1 were stained with TRPV2 primary antibody (**A**), or primary antibody omitted controls (**B**). Scale bars 25 μm. Confocal calcium imaging was carried out on PBEC_1 to investigate the functional expression of TRPV2 channels. Exemplar responses from 3 PBECs are shown with the addition of cannabidiol (CBD) and tranilast (TRN) (black arrow at 25 s) and addition of CBD alone (black arrow at 500 s) (**C**). Representative pseudo-coloured images showing PBEC responses to CBD ± tranilast (**D**). Peak responses (**E**) and area under curve of responses (**F**) of CBD ± tranilast were analysed. Mean with SEM, *N* = 91 individual PBECs. Wilcoxon matched-pairs signed rank test, ***P < 0.001

TRPV2 gene expression was confirmed by qPCR (Table E2) and TRPV2 protein expression was determined by immunocytochemistry (Fig. 3a and b). Similar TRPV2 protein expression data with alternative TRPV2 antibodies is available in the online supplement (supplementary Fig. 3). To study the functionality of TRPV2 in PBECs, confocal calcium imaging experiments were performed with the TRPV2 agonist CBD and the TRPV2 inhibitor tranilast [1 µM]. At present, there is a lack of specific TRPV2 pharmacological agonists, and as CBD can also act on cannabinoid receptors, we took a precautionary approach by undertaking all experiments in the presence of CB_1_ and CB_2_ receptor antagonists to ensure the specificity of CBD responses to TRPV2 (further detail is available in the online supplement). Stimulation of PBECs with CBD in the presence of tranilast prevented a rise in intracellular calcium; however, upon washout of tranilast, CBD evoked a robust rise in intracellular calcium (Fig. 3c). Representative CBD ± tranilast responses are visible with pseudo-coloured images (Fig. 3d). Tranilast significantly decreased peak (*P* < 0.001) and area under curve (*P* < 0.001) CBD responses (1.32 ± 0.06 and 468 ± 8.36 respectively) compared to CBD responses in the absence of tranilast (2.49 ± 0.46 and 884 ± 136.0 respectively) indicating inhibition of TRPV2 by 1 µM tranilast (Fig. 3f). Additional functional TRPV2 responses in PBECs to the agonist probenecid are presented in the online supplement (supplementary Fig. 4).

### TRPV2 expression in human bronchial epithelium

**Fig. 4:**
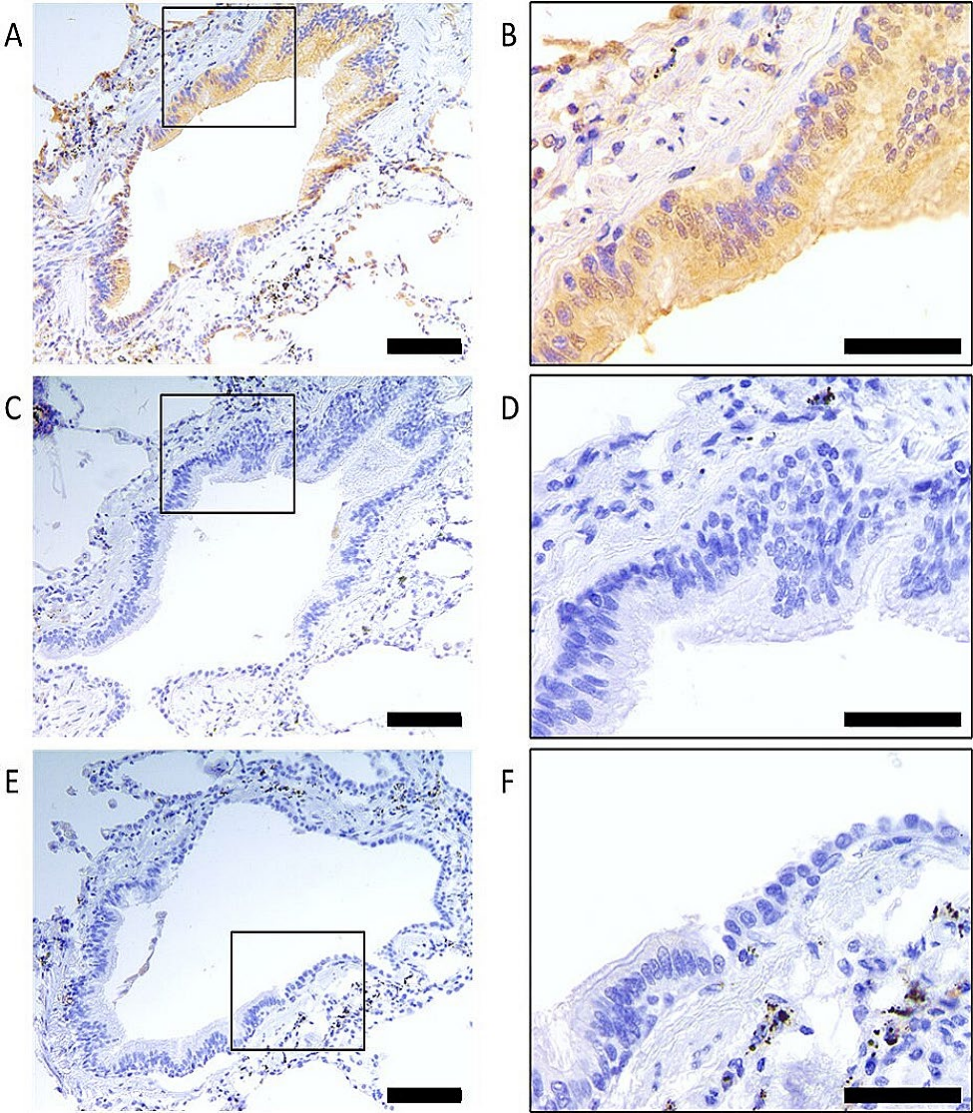
TRPV2 Expression in Distal Airway Human Lung Sections. Tissue sections were stained with TRPV2 antibody (**A** & **B**), rabbit IgG control (**C** & **D**), no primary antibody controls (**E** & **F**). Scale bars 100 μm (**A**, **C** & **E**) or 50 μm (**B**, **D** & **F**)

The bronchial epithelium of distal human lung sections stained positively for TRPV2 (Fig. 4a and b). IgG isotype control (Fig. 4c and d) and primary antibody omitted control (Fig. 4e and f) were free from nonspecific staining. Positive staining of the bronchial epithelium in these sections was observed with the epithelial marker cytokeratin (supplementary Fig. 5).

### Attenuation of ATP release from PBECs with TRPV2 pharmacological inhibition and siRNA knockdown

**Fig. 5:**
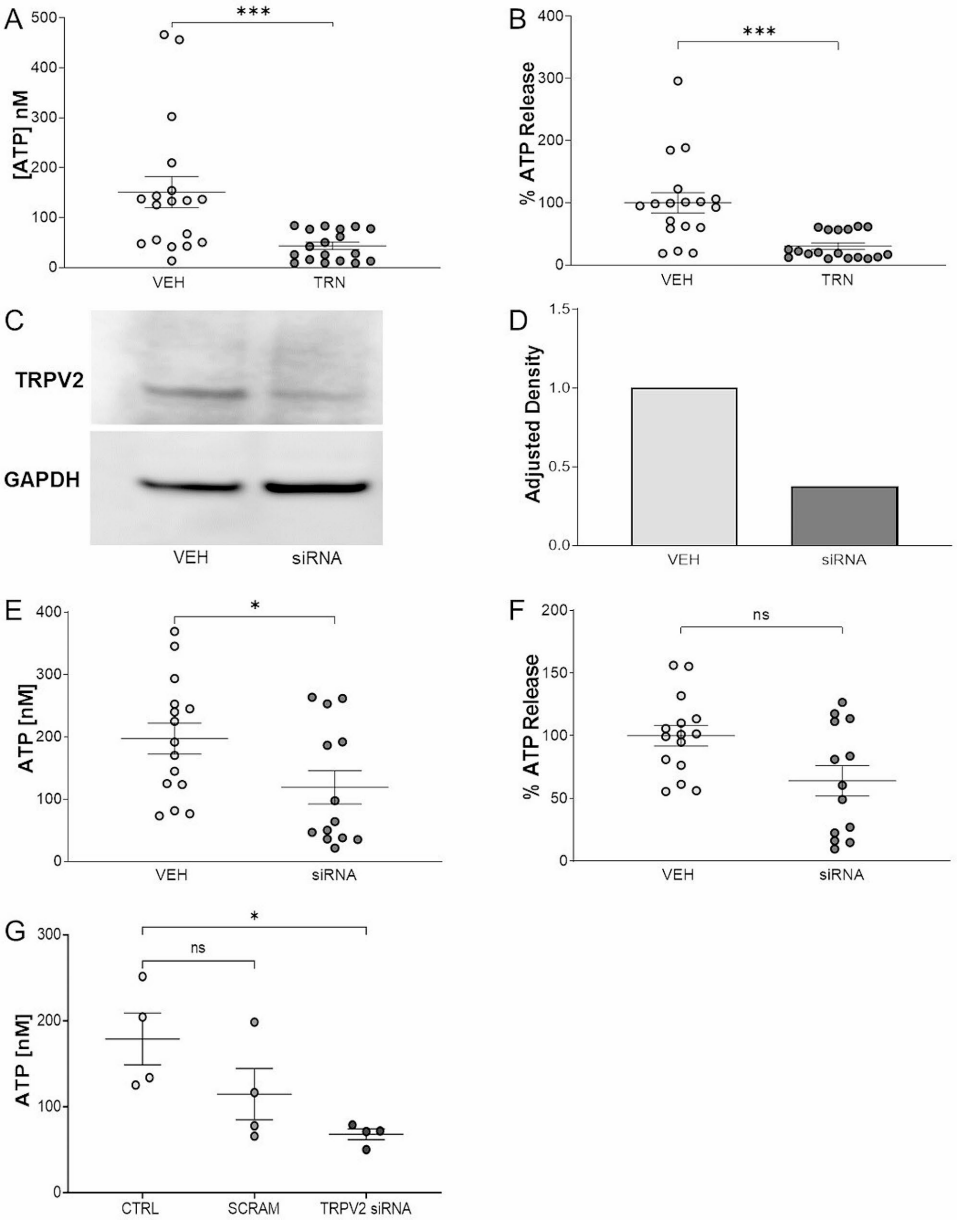
TRPV2 Mediates ATP Release in Fluid Shear Stress Stimulated Primary Bronchial Epithelial Cells (PBECs). ATP release from control (0 CPM) and fluid shear stress (FSS) stimulated PBEC_1 (100 CPM) treated with tranilast (TRN), or vehicle (VEH) was measured by ATPlite assay (**A**). Results were presented as percentage ATP release normalised to VEH (**B**). Mean with SEM, *N* = 3 independent experiments. Mann-Whitney test, ***P < 0.001. (**A** & **B**). TRPV2 expression in PBEC_2 transfected with 5 nM TRPV2 siRNA or transfection reagent vehicle (VEH) and exposed to fluid shear stress (FSS) at 100 CPM was measured by western blot 48 h post transfection. GAPDH was used as a loading control on the same membrane (**C**) (Full length blot available in the online supplement (supplementary Fig. 6a and 6b)). Semiqualitative densitometry analysis of TRPV2 band and GAPDH loading control band on the same membrane (**D**). ATP release from FSS stimulated PBEC_1 and PBEC_2 (100 CPM) transfected with 5 nM TRPV2 siRNA (siRNA) or transfection reagent vehicle (VEH) was measured 48 h post transfection (**E**). Results were presented as percentage ATP release normalised to VEH (**F**). Mean with SEM, *N* = 3 independent experiments. Mann-Whitney test *P < 0.05 (**E**) and *P* = 0.08 (**F**). ATP release from FSS stimulated PBEC_1 (100 CPM) transfected with 5 nM scrambled control (SCRAM) or TRPV2 siRNA (siRNA) was measured 48 h post transfection. Mean with SEM, *N* = 2 independent experiments. Kruskal-Wallis test with Dunn’s multiple comparisons test, *P < 0.05 and *P* = 0.31 (**G**)

The TRPV2 inhibitor tranilast and TRPV2 siRNA knockdown were used to assess the role of TRPV2 in ATP release from FSS stimulated PBECs. Maximal ATP release observed with 100 CPM FSS was significantly decreased (*P* < 0.001) by treatment with tranilast (vehicle: 159 ± 17.49 nM, tranilast: 25.08 ± 5.1 nM) (Fig. 5a), equivalent to a 69.6% decrease (*P* < 0.001) in ATP release (Fig. 5b). Treatment of PBECs with tranilast and FSS did not result in a decrease of cell viability as measured by MTT assay (supplementary Fig. 2c).

A modest decrease in TRPV2 protein expression was achieved using 5 nM siRNA knockdown and visualised by western blot (Fig. 5c) with the relative adjusted density of TRPV2 and GAPDH loading control semi-quantified by densitometry (vehicle: 1, siRNA: 0.38) (Fig. 5d). Maximal ATP release observed with 100 CPM FSS showed a decreasing trend (*P* < 0.05) with TRPV2 siRNA knockdown in PBECs (vehicle: 197 ± 24.52 nM, siRNA: 119 ± 26.85 nM) (Fig. 5e), equivalent to a 35.82% decrease in ATP release (Fig. 5f).

## Discussion

We have demonstrated that ATP is released from mechanically-stimulated human airway epithelial cells and for the first time, provide evidence of ex-vivo TRPV2 expression, localised to the bronchial epithelium in human lung tissue. Moreover, through pharmacological inhibition of TRPV2 with tranilast or using TRPV2 siRNA knockdown we observed significant attenuation of ATP release elicited by mechanical stimuli. We propose that TRPV2 is an important regulatory ion channel responsible for ATP release from the airway epithelium following exposure to mechanical stimuli. Repeated coughing, in conditions such as COPD and chronic cough, is likely to exert mechanical stress on the airway walls with resulting extracellular ATP accumulation in the lung, creating a positive feedback loop for persistent coughing.

While TRPV2 mRNA expression has been reported in human lung parenchyma [17], this is the first report of TRPV2 protein expression in human bronchial epithelium. TRPV2 signalling has previously been investigated using CBD in large human lung cell carcinoma and adenocarcinoma cell lines, but this study did not demonstrate expression of TRPV2 gene or protein [20]. The expression of TRPV2 in the human airway epithelium is relevant as epithelial cells may be readily activated by the compressive and shear forces exerted on the airways during bouts of coughing. Such a role could be considered comparable to mechanical activation of TRPV2 in endothelial cells and cardiomyocytes under dynamic physiological mechanical forces [18, 19].

Cough consists of deep inspiration followed by forced rapid expiration with compression and narrowing which exerts complex physical forces on the airway [21]. We used two means of eliciting mechanical stress on airway epithelial cells based on previously published methodology [4]. We recognise that our approach serves as a proxy for what may occur in vivo but an established physiologically gold standard is currently lacking. We observed that CFSS or FSS alone elicited comparable levels of ATP release from bronchial epithelial cells. Using the FSS method, we observed a dose-dependent relationship between shear stress stimulus intensity and ATP concentration, similar to that reported by Tarran et al. [22]. The FSS applied to cells in our study at 50 and 100 CPM was estimated to be 0.18 dynes/cm^2^ and 0.41 dynes/cm^2^ respectively, similar to tidal breathing induced shear stress, estimated to range between 0.19 and 0.73 dynes/cm^2^ dependent on airway generation [22]. In contrast, we found no evidence of increased IL-8 or MMP-13 release using FSS stimulation despite reports that IL-8 release or MMP activity is increased with stretching mechanical forces or compressive stress respectively [7, 8], indicating that ATP release occurs readily with FSS stimulation whereas the release of other inflammatory mediators does not. Interestingly, Yegutkin et al. (2000) found that shear stress treatment increased the extracellular activity of ATP degrading soluble ATPases and AMP degrading 5’-nucleotidases, suggesting that the increased ATP levels (in the current study) are unlikely to have arisen via reduced ATP degradation [23].

We also report novel findings concerning the mechanisms responsible for mechanical stress elicited ATP release from the airway epithelium; the expression and functional role of TRPV2 in PBECs has not previously been documented and both pharmacological inhibition of TRPV2 with tranilast and siRNA knockdown suggests a role for TRPV2, at least in part, in FSS evoked ATP release. Tranilast, was originally licensed for use in allergic disorders such as rhinitis, asthma and atopic dermatitis [24]. Tranilast suppresses the transcription factor SMAD4 and accordingly inhibits the expression or action of the transforming growth factor beta pathway, inhibits the activity of the sepiapterin reductase enzyme and has anti-proliferative effects in addition to acting as a TRPV2 channel inhibitor; nonetheless, the mechanism of action by which tranilast inhibits TRPV2 remains to be determined [25–28]. A modest reduction of TRPV2 protein expression in PBECs achieved by TRPV2 siRNA intervention, however a functional effect was still observed as decreased ATP release was noted with decreased TRPV2 expression. Thus, the results observed with TRPV2 siRNA knockdown corroborated the results obtained from tranilast inhibition indicating a role for TRPV2 in ATP release from mechanically stressed PBECs. To add further evidence for the role of TRPV2 in ATP release from PBECs, additional pharmacological experiments linking TRPV2 activation with ATP release from PBECs are presented in the online supplement (supplementary Fig. 7).

We acknowledge that TRPV2 is not likely to be the sole mechanosensitive receptor responsible for ATP release in PBECs as a number of primary or auxiliary ATP release routes may be involved, contingent on the type of mechanical stress applied. As ATP release mechanisms are related to the type of mechanical stimulus, volume-regulated anion channels and pannexins are unlikely to be major contributors to FSS dependent ATP release as both of these channels are more likely to contribute to either osmotic or strain mechanical stimuli [13]. Hemichannels, maxi-anion channels or vesicular ATP release routes are likely to contribute to cellular ATP release with FSS stimulation in addition to osmotic or strain mechanical stimuli [13].

In conclusion, this data provides additional insight into the mechanism underlying mechanically stimulated ATP release from the airway epithelium. We report a mechanosensitive role for TRPV2 and provide evidence for its expression in human airway epithelium. Our findings provide some support for the notion that cough begets cough, whereby compressive and shear mechanical stimulation of the airway, potentially through coughing, causes extracellular ATP release from epithelial cells which can act as an agonist for P2 × 3 receptors expressed on airway sensory nerves to evoke further coughing.

### Electronic supplementary material

Below is the link to the electronic supplementary material.

Supplementary Material 1

Supplementary Material 2

## Data Availability

The data supporting the conclusions of this article are included within the article (and its online supplement).

## Declarations

### Declarations

This study was not a clinical trial.
