# Supplementary material for: TRPV2 modulates mechanically Induced ATP Release from Human bronchial epithelial cells
Source: Respir Res. 2024 Apr 27;25:188. doi: 10.1186/s12931-024-02807-0 (PMC11056070; doi:10.1186/s12931-024-02807-0)
Supplement: Supplementary file 1 — Supplementary Material 1 [file 12931_2024_2807_MOESM1_ESM.docx]

**TRPV2 Modulates Mechanically Induced ATP Release from Human Bronchial Epithelial Cells.**

Orla M. Dunne^1^, S. Lorraine Martin^2^, Gerard P. Sergeant^3^, Daniel F. McAuley^1^, Cecilia M. O’Kane^1^, Button B^4^, Lorcan P. McGarvey^1^, Fionnuala T. Lundy^1^.

1. Wellcome-Wolfson Institute for Experimental Medicine, School of Medicine, Dentistry and Biomedical Sciences, Queen's University Belfast, Belfast, UK.
2. School of Pharmacy, Queen's University Belfast, Belfast, UK.
3. Smooth Muscle Research Centre, Dundalk Institute of Technology, Dundalk, Co. Louth, Ireland.
4. Department of Biochemistry and Biophysics, University of North Carolina at Chapel Hill, North Carolina, United States.

Corresponding author:

Lorcan McGarvey, Wellcome-Wolfson Institute for Experimental Medicine, School of Medicine, Dentistry and Biomedical Sciences, 97 Lisburn Road, Belfast BT9 7BL, UK. [l.mcgarvey@qub.ac.uk](mailto:l.mcgarvey@qub.ac.uk)

**Drugs and Solutions**

Hanks balanced salt solution (HBSS) contained 140 mM sodium chloride (Thermo Fisher Scientific, Paisley, UK), 5 mM potassium chloride (VWR BDH Chemicals, Leicestershire, UK), 2 mM calcium chloride (Sigma Aldrich, Burlington, USA), 1 mM magnesium chloride (Sigma Aldrich), 10 mM HEPES free acid (Fluorochem, Glossop, UK) and 5 mM D-glucose (Thermo Fisher Scientific) (pH 7.4). Phosphate buffered saline (PBS) contained 145 mM sodium chloride (Thermo Fisher Scientific), 7.47 mM disodium hydrogen phosphate (Thermo Fisher Scientific) and 2.5 mM sodium phosphate monobasic (Acros Organics, Geel, Belgium) (pH 7.4). 4% paraformaldehyde (PFA) solution contained PFA (Sigma Aldrich) dissolved in distilled water and further diluted in 2X PBS (pH 7.4). Sodium citrate antigen retrieval solution contained 10 mM tri-sodium citrate dehydrate (Sigma Aldrich) supplemented with 0.05% Tween (pH 6.0). Bovine serum albumin (BSA) blocking solution contained 3% BSA (Sigma Aldrich) in 1X PBS. Radioimmunoprecipitation assay (RIPA) buffer contained 50 mM trizma base (Sigma Aldrich), 150 mM sodium chloride (Thermo Fisher Scientific), 1% IGEPAL (Sigma-Aldrich), 0.25% sodium deoxycholate (Sigma-Aldrich), 1 mM EDTA (Thermo Fisher Scientific) (pH 7.4). Tris buffered saline (TBS) contained 200 mM trizma base (Sigma Aldrich) and 1.5 M sodium chloride (Thermo Fisher Scientific) (pH 7.6). CBD, tranilast, JTE907 and AM251 were purchased from Tocris (Abingdon, UK) and reconstituted in dimethyl sulfoxide (DMSO) (Honeywell, Charlotte, USA).

**Table E1. Primary Bronchial Epithelial Cell (PBEC) Sample Number and Harvesting Method with Donor Gender, Pack Years, FEV_1_, and Obstructive Phenotype.**

| **PBEC Sample Number** | **Harvesting Method** | **Gender** | **Smoking status (Pack Years)** | **FEV_1_** | **FEV_1_**  **(% predicted)** | **Obstructive Phenotype** |
| --- | --- | --- | --- | --- | --- | --- |
| PBEC_1 | Bronchoscopy | Female | Non-smoker  (N/A) | 2.2 | 79 | Refractory chronic cough, non-obstructive lung disease |
| PBEC_2 | Bronchoscopy | Male | Smoker  (40 pack years) | 2.28 | 83 | COPD |
| PBEC_3 | Bronchoscopy | Male | Non-smoker  (N/A) | 2.98 | 108 | Healthy |
| PBEC_4 | Bronchial brush from ex-vivo lung tissue | Male | Smoker  (Not available) | Information not available | Information not available | Information not available |
| PBEC_5 | Bronchoscopy | Female | Smoker  (40 pack years) | 1.03 | 45 | COPD |

**Measurement of ATP Release from Mechanically Stimulated PBECs**

PBECs were grown in 6-well plates with epithelial cell growth medium (PromoCell, Heidelberg, Germany) to 70% – 90% confluency and exposed to mechanical stimulation for 30 minutes. ATP in the conditioned culture medium of PBECs was measured immediately after mechanical stimulation by ATPlite luciferin-luciferase assay with a minor modification to the manufacturer’s instructions. Briefly, reagents were reconstituted, and ATP standards were produced as per the manufacturers protocol. Mammalian cell lysis solution was added to harvested culture medium or to ATP standards in a white 96-well microplate (Thermo Scientific) and the reaction was incubated for 5 minutes at room temperature. Substrate solution was added to wells and the reaction was incubated for a further 5 minutes before being read on a Varioskan Lux plate reader with an endpoint luminescence reading.

**Measurement of Inflammatory Mediators Produced by Mechanically Stimulated PBECs**

PBECs were grown in 6-well plates with epithelial cell growth medium (PromoCell) to 70% – 90% confluency and exposed to mechanical stimulation for 30 minutes. IL-8 was measured in the conditioned culture medium of PBECs by ELISA (R&D systems, Abingdon, UK), 24 hours after mechanical stimulation, according to the manufacturer’s instructions. MMP-13 was measured in the cell lysate of PBECs 24 hours after mechanical stimulation, using a fluorescence resonance energy transfer assay (SensoLyte ® Plus 520; AnnaSpec, Fremont, USA), according to the manufacturer’s instructions.

**TRPV2 Gene Expression in Primary Bronchial Epithelial Cells (PBECs) Determined by qPCR**

Ribonucleic acid (RNA) was harvested from PBECs using the Maxwell RNA isolation kit (Promega, Wisconsin, USA) and quantified using a Nanodrop (Thermo Fisher Scientific). The SuperScript VILO cDNA synthesis kit (Thermo Fisher Scientific) was used to synthesise cDNA from RNA samples. qPCR reaction mixtures were made up with TaqMan universal master-mix containing UNG (Thermo Fisher Scientific) and predesigned TaqMan primers (Thermo Fisher Scientific) for TRPV2 and reference genes glucuronidase beta (GUSB) and beta-2-mircoglobulin (B2M). qPCR was carried out with the Stratagene PCR instrument (Agilent Technologies, California, USA). Data is presented as cycle of quantification (Cq) values. qPCR investigating TRPV2 expression was carried out in PBECs originating from 3 donors PBEC_1, PBEC_2 or PBEC_5.

**TRPV2 Immunocytochemistry of PBECs**

PBECs were fixed with 4% PFA for 10 minutes at room temperature and residual PFA was quenched with PBS supplemented with 0.1 M glycine (VWR BDH Chemicals) for 20 minutes. Permeabilization of PBECs was carried out with PBS supplemented with 0.05% Triton 100-X for 20 minutes at room temperature. PBECs were incubated with 10% normal goat serum (Sigma Aldrich) supplemented with 0.01% Triton 100-X for 1 hour to block non-specific binding sites. PBECs were incubated with TRPV2 primary antibody (LS-B4211, LS-Biosciences, Seattle, USA) overnight at 4°C. PBECs were incubated with a goat anti- rabbit Alexa Fluor 488 (Invitrogen, Waltham, USA) secondary antibody for 1 hour at room temperature. 4’,6 diamidino-2-phenylindole (DAPI) (Invitrogen) was used to counterstain nuclei and mount PBECs to microscope slides. Negative controls omitted the addition of primary antibodies.

**Intracellular Calcium Measurements in PBECs**

Calcium mobilisation was measured in PBECs grown on 35 mm glass bottom dishes (WillCo wells, Amsterdam, Netherlands) by confocal calcium imaging. PBECs were loaded with 1 µM Fluo-4 AM (Thermo Fisher Scientific) in HBSS for 60 minutes at 37°C and mounted on the stage of a SP5 inverted microscope (Leica microsystems, Wetzlar, Germany).

At present there is a lack of selective human TRPV2 pharmacological agonists; thus, CBD was selected for use in this study as a TRPV2 agonist. Unlike the TRPV2 agonist Aminoethoxydiphenyl borate (2-APB) which exhibits species dependent activation of rat and mouse but not human TRPV2 [27], there is evidence of CBD binding and activating human TRPV2 [27–29]. Several recent studies demonstrate that CBD may act as an antagonist of cannabinoid receptor type 1 (CB_1_) and cannabinoid receptor type 2 (CB_2_) receptors, and CBD can act as an inverse agonist of the CB_2_ receptor; whereas another paper reports that CBD acts as a partial agonist at the CB_2_ receptor[30–32]. Due to conflicting information relating to the effect of CBD on CB_1_ and CB_2_ receptors we replicated a precautionary approach undertaken by Eubler et al., to carry out all experiments in the presence of CB_1_ and CB_2_ antagonists to ensure the specificity of CBD responses to TRPV2. Thus, PBECs were maintained at 37°C by constant perfusion with pre-warmed HBSS supplemented with CB_1_ and CB_2_ receptor antagonists AM251 [80 nM] and JTE907 [36 nM] respectively to prevent unwanted activation of CB_1_ and CB_2_ receptors by CBD. Likewise, tranilast was selected for use as a TRPV2 inhibitor in this study as it is widely used for this purpose [19, 23, 33].

XYT stacks were acquired with a X20 objective (HC PL FLUOTAR 20.0x0.50 DRY) at 7 frames per second using Leica Application Suite Advanced Fluorescence (LAS AF) software (Leica microsystems). Pharmacological compounds were delivered using an automated drug delivery system (ALA Scientific Instruments, Farmingdale, USA) and a perfusion pencil with a 250 µm removable tip (Digitimer, Welwyn, UK). PBECs were stimulated with 1 µM CBD ± 1 µM tranilast. For quantitative analysis, each cell in a field of view was selected as a region of interest (ROI) with LAS AF software and the fluorescence over fluorescence at rest (F/F_0_) for each ROI was calculated.

**Immunohistochemistry on Human Lung Sections**

Human lung tissue was obtained with ethical approval from the International Institute for the Advancement of Medicine and the QUB School of Medicine, Dentistry and Biomedical Science Research Ethics Committee (REF 14/08). 5 µM sections of formalin-fixed paraffin embedded human lung tissue containing intralobular bronchioles were deparaffinised and rehydrated with clearene and a series of decreasing ethanol concentrations respectively. Heat induced epitope retrieval was carried out with sodium citrate antigen retrieval solution. Slides were blocked using consecutive room temperature incubations of Bloxall blocking solution (Vector laboratories, Burlingame, USA) for 15 minutes, 3% BSA blocking buffer for 15 minutes, and 10% goat serum (Sigma Aldrich) for 1 hour. TRPV2 primary antibody (LS-B4211, LS-Biosciences) and cytokeratin primary antibody (M3515, Dako, Stockport, UK) were diluted 1:100 in 10% goat serum and applied to sections overnight at 4°C. Rabbit (TRPV2) and mouse (cytokeratin) HRP conjugated secondary antibodies supplied at working concentration (goat anti-rabbit; MP-7451 Vector Laboratories or goat anti-mouse; MP-7452 Vector Laboratories) were applied to sections for 1 hour at room temperature. Immunoglobulin G (IgG) isotype controls and negative controls with the omission of primary antibodies were carried out in parallel to the staining procedure. 3,3′-Diaminobenzidine (DAB) (Vector Laboratories) reagent was applied to sections for 4 to 6 minutes, and the sections were counterstained with haematoxylin before being dehydrated with an increasing ethanol concentration series and cleared with clearene. DPX mounting medium (Sigma Aldrich) was used to mount coverslips to slides.

**Semi-quantitative Analysis of TRPV2 Expression in PBEC with siRNA Transfection**

PBEC_2 were transfected with 0 or 5 nM TRPV2 siRNA (Qiagen) using HiPerfect transfection reagent (Qiagen) 24 hours after seeding onto 6-well plates. PBECs were treated with FSS mechanical stimulation 48 hours after transfection and lysed with RIPA buffer. Total protein concentration was determined using a bicinchoninic acid (BCA) protein assay kit (Thermo Fisher Scientific) and samples normalised to 10 µg and loaded to a NuPAGE 4-12% Bis-Tris gel (Thermo Fisher Scientific) and run with the XCell SureLock rig and NuPAGE MES SDS running buffer (Life Technologies, Thermo Fisher Scientific). Proteins were transferred from the gel to a nitrocellulose membrane with NuPAGE Transfer Buffer (Life Technologies, Thermo Fisher Scientific). The membrane was blocked in 5% BSA in 0.05% TBS-Tween for 1 hour at room temperature. The membrane was probed with TRPV2 primary antibody (72-kDa, LS-B4211, LS-Biosciences) diluted to the working concentration (1:500) in the block solution overnight at 4°C. Goat anti-rabbit IgG peroxidase conjugated to HRP (AP132P, Sigma-Aldrich) was diluted to the working concentration (1:750) in the block solution and applied to the membrane for 1 hour at room temperature. The membrane was developed with Clarity Western ECL Substrate (Bio-Rad Laboratories, Hercules, California, United States) and imaged with a Syngene G: Box. The membrane was stripped with Restore Western Blot Stripping Buffer (Thermo Fisher Scientific) and re-probed with GAPDH monoclonal antibody (37-kDa, AM4300, Thermo Fisher Scientific) diluted to working concentration (1:1000) for 1 hour at room temperature. Goat anti-mouse IgG peroxidase conjugated to HRP (AP124P, Sigma-Aldrich) was diluted to working concentration (1:5000) in the block solution and applied to the membrane for 1 hour at room temperature. The membrane was developed and imaged as previously described. Membranes were analysed using densitometric analysis with FIJI software (National Institute of Health, USA, Version 1.54).

**Calculations of *in vitro* fluid shear stress (FSS) on PBEC cultures**

To calculate shear, we assumed that the culture media had an equal density to water and the shear evoked by each revolution was:

$\tau$shear *= μ⋅* $\left( \frac{dy}{du} \right)$

Where *μ* is the dynamic viscosity of the fluid (10^-3^ Pa·s), $\frac{dy}{du}$ is the rate of change of velocity in the transverse direction to flow (i.e., shear rate) where $dy$ denotes velocity calculated by dividing the distance the platform moved in one cycle by the speed taken to move one cyle (25 CPM: 0.01739130 m/s, 50 CPM: 0.03539823 m/s and 100 CPM: 0.081632653 m/s), and $du$ represents average liquid layer height (0.002 m).

$\tau$shear 25 CPM = 10^-3^ Pa·s *⋅*$\left( \frac{0.01739130 m/s}{0.002 m} \right)$ = 0.008695652 Pa = 0.09 dynes/cm^2^

$\tau$shear 50 CPM = 10^-3^ Pa·s *⋅*$\left( \frac{0.03539823m/s}{0.002 m} \right)$ = 0.017699115 Pa= 0.18 dynes/cm^2^

$\tau$shear 100 CPM = 10^-3^ Pa·s *⋅*$\left( \frac{0.081632653 m/s}{0.002 m} \right)$ = 0.040816327 = 0.41 dynes/cm^2^

**Table E2. TRPV2 Gene Expression in Primary Bronchial Epithelial Cells (PBECs) Determined by qPCR.**

| Gene | PBEC | Mean Cq of technical replicates | SEM |
| --- | --- | --- | --- |
| TRPV2 | PBEC_1 | 34.12 | 0.32 |
|  | PBEC_2 | 32.14 | 0.28 |
|  | PBEC_5 | 31.00 | 0.22 |
| GUSB | PBEC_1 | 23.79 | 0.08 |
|  | PBEC_2 | 24.38 | 0.07 |
|  | PBEC_5 | 24.22 | 0.13 |
| B2M | PBEC_1 | 17.44 | 0.17 |
|  | PBEC_2 | 19.16 | 0.18 |
|  | PBEC_5 | 19.06 | 0.16 |

#
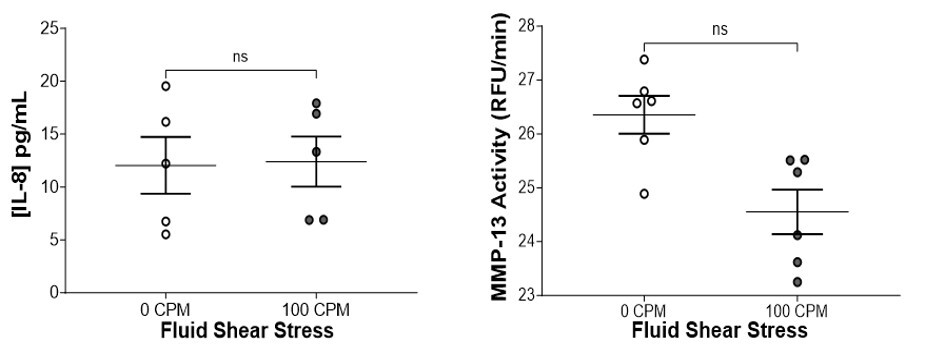
Supplementary Figures

**Figure E1. Measurement of Inflammatory Factors in Fluid Shear Stress Stimulated Primary Bronchial Epithelial Cells (PBECs).** IL-8 release in control (0 CPM) and fluid shear stress (100 CPM) stimulated PBEC_1 was quantified by ELISA (R&D systems). Mean with SEM, N=3 independent experiments. Mann-Whitney test, P>0.05 (A). MMP-13 activity in control (0 CPM) and fluid shear stress (100 CPM) stimulated PBEC_1 was quantified by fluorescence resonance energy transfer assay (SensoLyte® Plus 520; AnnaSpec). Mean with SEM, N=3 independent experiments. Mann-Whitney test, P>0.05 (B).

**
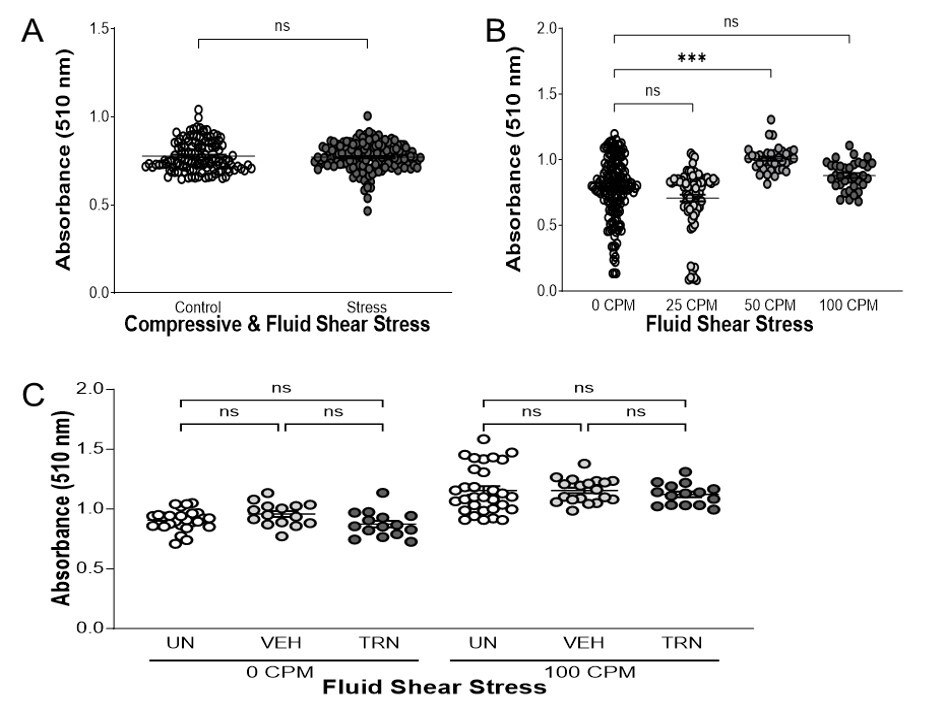
**

**Figure E2. Cell Viability of Mechanically Stimulated Primary Bronchial Epithelial Cells (PBECs) was Assessed using a MTT Assay.** A MTT assay was carried out as per the manufacturer’s instructions to determine the effects of compressive and fluid shear stress stimulation on PBEC_1 cell viability. Mean with SEM, N=3 independent experiments. Mann-Whitney test, P>0.05 (A). A MTT assay was carried out as per the manufacturer’s instructions to determine the effects of fluid shear stress at 0 CPM (control), 25 CPM, 50 CPM or 100 CPM intensities on PBEC_1 cell viability. Mean with SEM, N=3 independent experiments. Kruskal-Wallis test with Dunn's multiple comparison test, ***P<0.001 (B). A MTT assay was carried out in control (0 CPM) or fluid shear stress (100 CPM) stimulated and untreated (UN) or treated with vehicle control (VEH) or tranilast (TRN) PBEC_1. Mean with SEM, N=3 independent experiments. Two-way ANOVA with Tukey’s multiple comparisons post-test, P>0.05 (C).


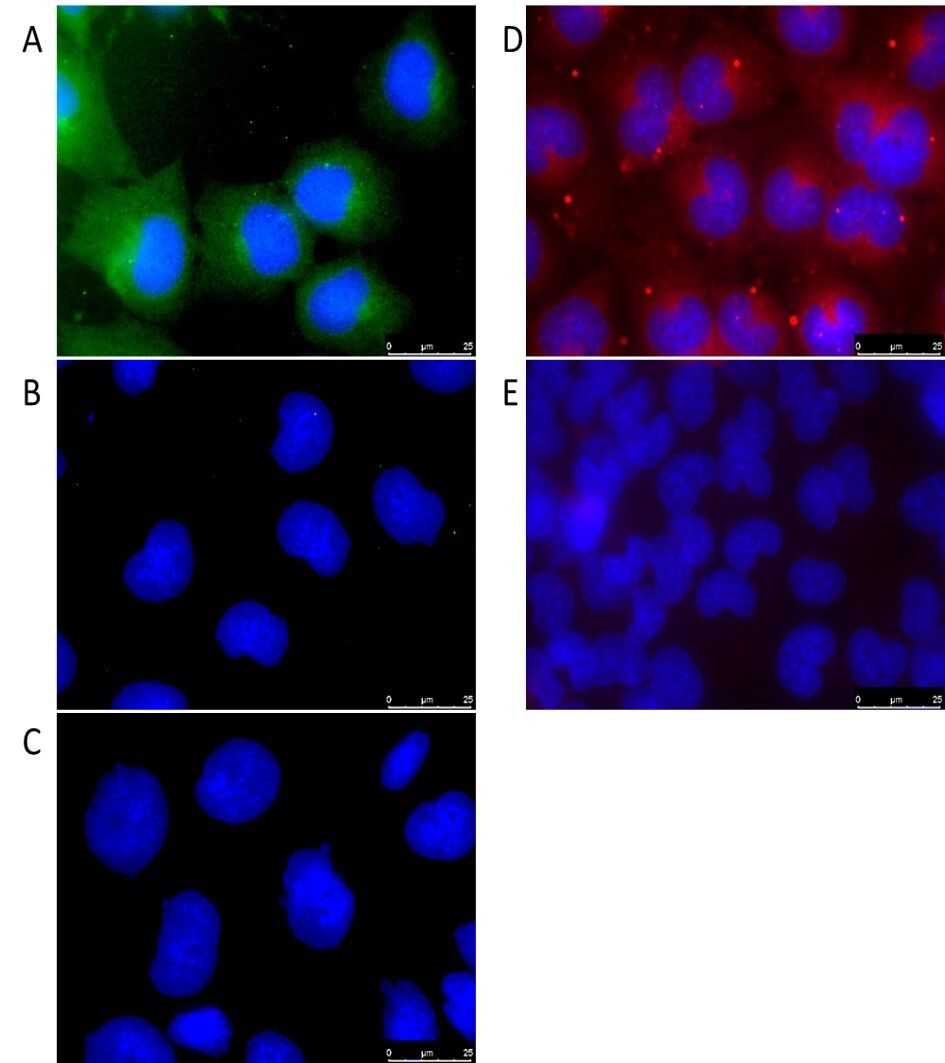


**Figure E3. TRPV2 Expression in Primary Bronchial Epithelial Cells (PBECs).** PBEC_1 stained positively for TRPV2 with Calbiochem PA1-18351 primary antibody (A). No staining was observed in the presence of the TRPV2 blocking peptide (B), or primary antibody omitted control (C). PBEC_1 stained positively for TRPV2 with Sigma SAB1101376 primary antibody (D). No staining was observed in primary antibody omitted control (E). Blocking peptide for Sigma antibody was not available. Scale bars 25 µm.


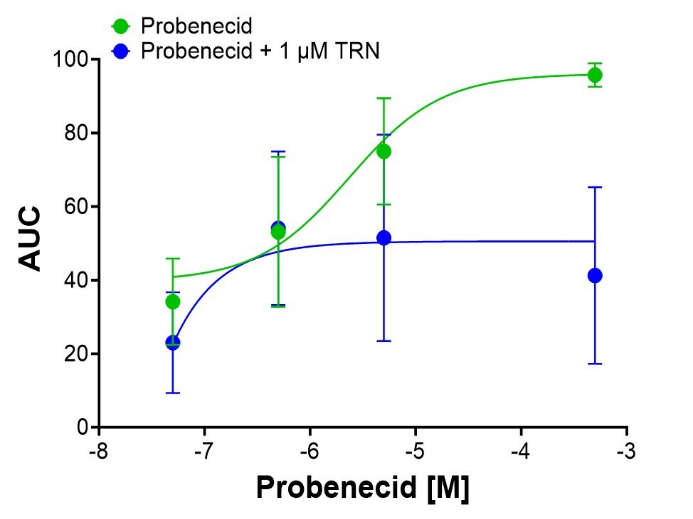


**Figure E4. Tranilast Inhibits Probenecid Responses in PBECs**. PBEC_1 responses to the TRPV2 agonist probenecid [0.05, 0.5, 5 and 500 µM] in the absence and presence of tranilast [1 µM] were evaluated by FURA 2-AM FLIPR assay. Mean with SEM, N=2 independent experiments.

**
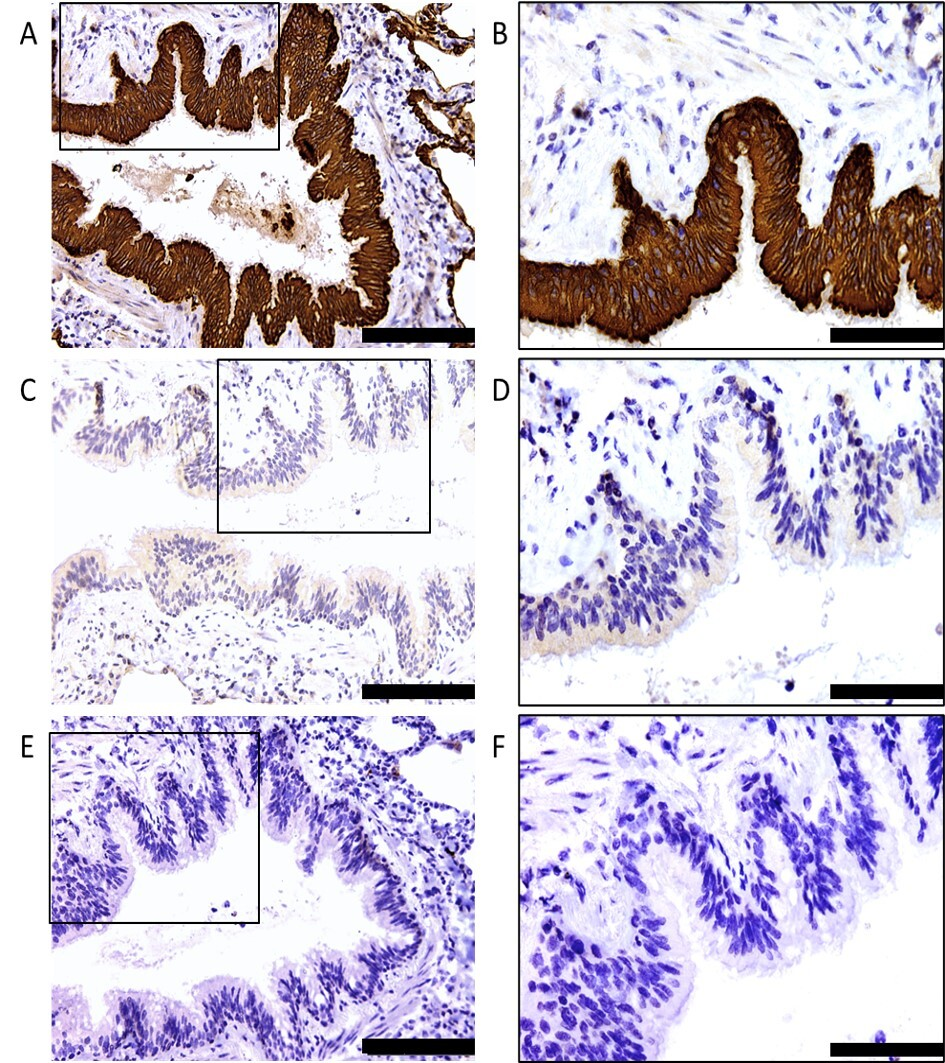
Figure E5. Cytokeratin Expression in Distal Airway Human Lung Sections.** Tissue sections were stained with cytokeratin antibody (A & B), mouse IgG control (C & D), no primary antibody controls (E and F). Scale bars 100 µm (A, C & E) or 50 µm (B, D & F).


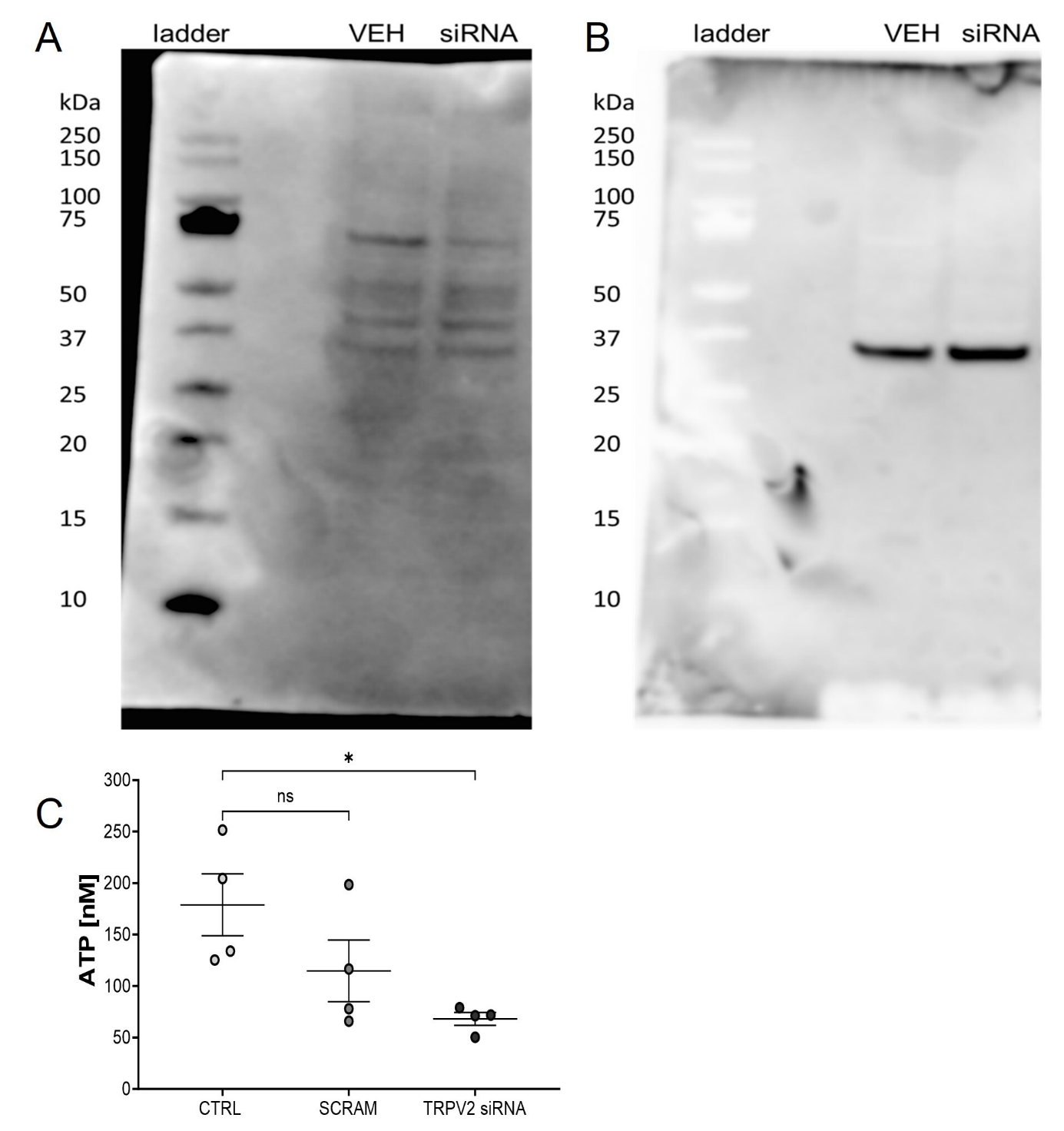
**Figure E6. Controls for siRNA Data Presented in Figure 5.** Full length western blot nitrocellulose membrane of PBEC samples transfected with 0nM TRPV2 siRNA (VEH) or 5nM TRPV2 siRNA (siRNA) probed with TRPV2 antibody (LS-B4211) (A). The membrane was stripped and re-probed with GAPDH antibody (AM4300) (B). For scrambled control experiments, PBEC_1 were transfected with 5 nM TRPV2 siRNA (Qiagen, NM_016113) or negative control siRNA (Qiagen, 1027281). PBECs were treated with FSS mechanical stimulation 48 hours after transfection and ATP was measured immediately by ATPlite luciferin-luciferase assay. Mean with SEM, N=2 independent experiments, each dot represents a single technical replicate. Kruskal-Wallis test with Dunn’s multiple comparisons test, *P<0.05 (C).


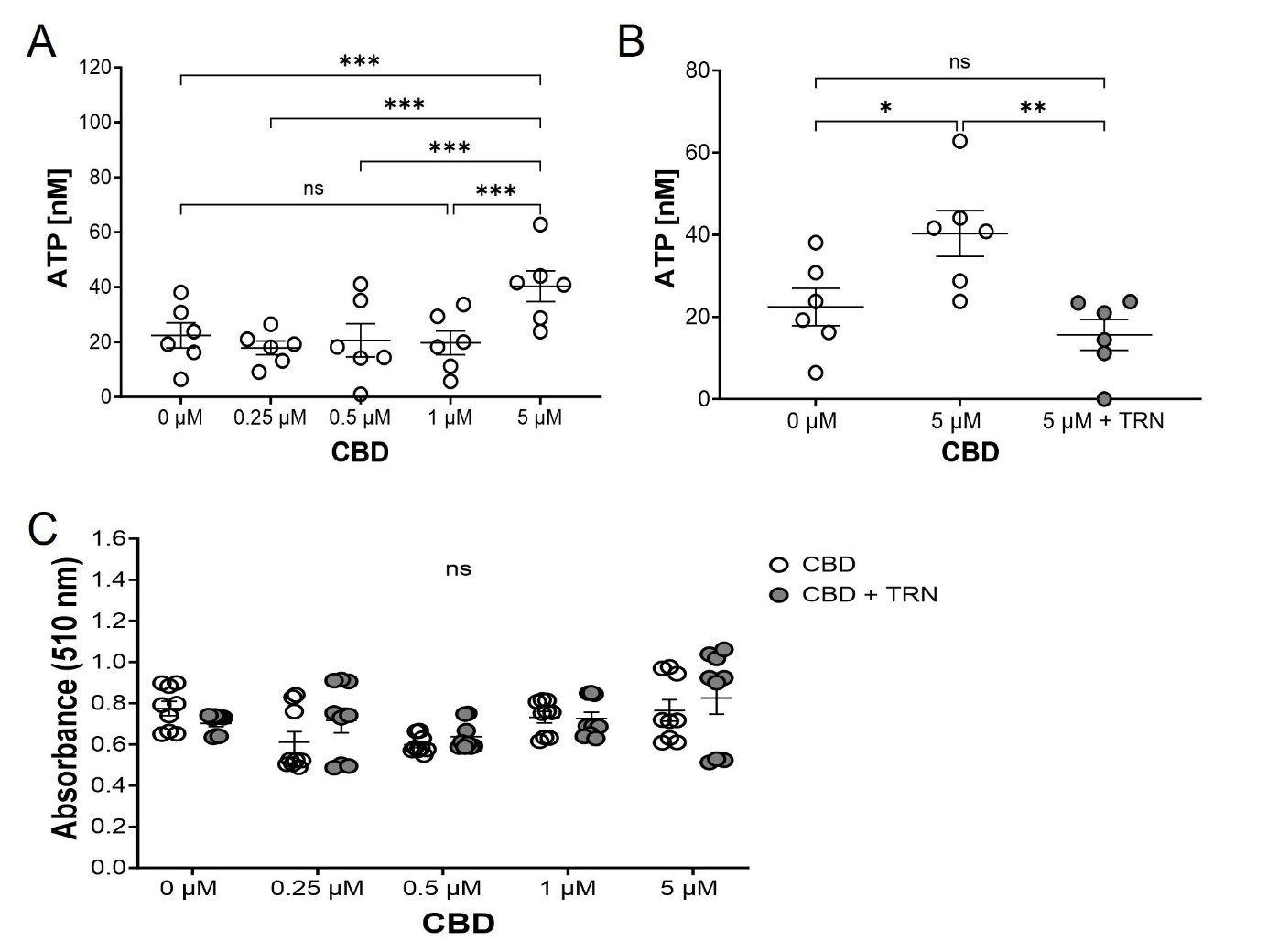


**Figure E7 Tranilast Inhibits CBD Evoked ATP Release from Primary Bronchial Epithelial Cells (PBECs).** ATP release from PBEC_1 treated with CBD [0, 0.25, 0.5,1 and 5 µM] was measured with the ATPlite luciferin-luciferase assay. Mean with SEM, N=3 independent experiments. Ordinary one-way ANOVA with Tukey’s multiple comparisons test, ***P<0.001 (A). ATP release from PBEC_1 treated with CBD [0 or 5 µM] or CBD and tranilast (TRN) [1 µM] was measured with the ATPlite luciferin-luciferase assay. Mean with SEM, N=3 independent experiments. Ordinary one-way ANOVA with Tukey’s multiple comparisons test, *P<0.05, **P<0.01 (B). The cell viability of PBEC_1 treated with CBD [0, 0.25, 0.5, 1 and 5 µM] ± tranilast (TRN) [1 µM] was measured by MTT assay. N=3 independent experiments. Two-way ANOVA with Dunnett’s multiple comparisons test (C).
